# EEG-Response Consistency across Subjects in an Active Oddball Task

**DOI:** 10.1371/journal.pone.0074572

**Published:** 2013-09-20

**Authors:** Yvonne Höller, Aljoscha Thomschewski, Jürgen Bergmann, Martin Kronbichler, Julia S. Crone, Elisabeth V. Schmid, Kevin Butz, Peter Höller, Eugen Trinka

**Affiliations:** 1 Department of Neurology, Paracelsus Medical University, Salzburg, Austria; 2 Neuroscience Institute & Center for Neurocognitive Research, Paracelsus Medical University, Salzburg, Austria; 3 Department of Psychology & Center for Neurocognitive Research, University of Salzburg, Salzburg, Austria; University of Bern, Switzerland

## Abstract

The active oddball paradigm is a candidate task for voluntary brain activation. Previous research has focused on group effects, and has largely overlooked the potential problem of interindividual differences. Interindividual variance causes problems with the interpretation of group-level results. In this study we want to demonstrate the degree of consistency in the active oddball task across subjects, in order to answer the question of whether this task is able to reliably detect conscious target processing in unresponsive patients. We asked 18 subjects to count rare targets and to ignore frequent standards and rare distractors in an auditory active oddball task. Event-related-potentials (ERPs) and time-frequency data were analyzed with permutation-t-tests on a single subject level. We plotted the group-average ERPs and time-frequency data, and evaluated the numbers of subjects showing significant differences between targets and distractors in certain time-ranges. The distinction between targets/distractors and standards was found to be significant in the time-range of the P300 in all participants. In contrast, significant differences between targets and distractors in the time-range of the P3a/b were found in 8 subjects, only. By including effects in the N1 and in a late negative component there remained 2 subjects who did not show a distinction between targets and distractors in the ERP. While time-frequency data showed prominent effects for target/distractor vs. standard, significant differences between targets and distractors were found in 2 subjects, only. The results suggest that time-frequency- and ERP-analysis of the active oddball task may not be sensitive enough to detect voluntary brain activation in unresponsive patients. In addition, we found that time-frequency analysis was even less informative than ERPs about the subject’s task performance. Despite suggesting the use of more sensitive paradigms and/or analysis techniques, the present results give further evidence that electroencephalographic research should rely more strongly on single-subject analysis because interpretations of group-effects may be misleading.

## Introduction

Voluntary brain activation is an important issue in contemporary research. The number of papers addressing this issue is boosted significantly by the scientific fields of ‘disorders of consciousness’ (DOC) and ‘brain computer interfaces’. The goals of these two fields overlap to some extent, and there are some research problems which have to be solved by both of them. One of these problems is the interindividual variance of response patterns to identical stimuli.

In brain computer interfaces, voluntary brain activation is used to communicate with a system solely by altering brain activity [1]. The problem of interindividual variance of response patterns is commonly solved by machine learning techniques. An algorithm is applied to a training set of sequences for each single subject to learn their typical response or responses (depending on the algorithm). This is known as the training phase. Following the training phase a classifier compares new sequences of brain activity to the learned typical response(s), and decides how to categorize the new sequence. Machine learning techniques are largely independent of interindividual variance as long as the features extracted from the training data allow a certain degree of freedom. For example, if one chooses 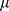-activity in the range of 10–15 Hz as such a feature in a motor imagery paradigm, those subjects with reactivity in frequency ranges below 10 and above 15 Hz will end up with low classification-accuracy rates.

In DOC voluntary brain activation is used to get an accurate distinction between subjects who do not have conscious awareness and subjects who are able to follow commands. Patients who are unable to follow commands behaviorally may be conscious but could mistakenly be diagnosed as being unconscious because clinical assessment is based on behavioral signs of consciousness. Therefore, the patients are confronted with simple commands which can be followed by thought [2]–[14]. To make research feasible for clinical practice it is necessary to provide recommendations for analyzing and statistically evaluating the data on a single-subject level. Group-level effects may not be concordant with effects in single subjects. Indeed, while most subjects show a desynchronization in the alpha range during motor imagery, there is a small number of subjects who show an alpha synchronization during this task [15].

Recently, several research groups have adopted single-subject statistics in electroencephalographic research of disorders of consciousness (e.g. [2], [4], [6], [7], [16]). In one of these studies, machine-learning techniques were applied for the assessment of patients with disorders of consciousness in a motor imagery task [4]. We believe that this may be the most promising approach to be used in clinical practice. However, even for machine-learning techniques it is important to know the features which best describe the individual response patterns. More concretely, the features for the classifier may be chosen so that they are invariant to interindividual differences in response patterns. To design accurate features it is necessary to get experience about the consistency of effects across subjects. In addition, it was shown that there are several statistical pitfalls in the interpretation of classification accuracies [17].

In the present study we examined the consistency of effects in an active oddball task. In this task subjects are asked to silently count a rare target sound and to ignore a frequent standard and a rare distractor. Such an experiment should produce a P3a and P3b component [18] in the event related potential (ERP). The distinction between P3a and P3b allows us to ascertain whether the subject counted the target or simply listened to the presented sequence of sounds. To apply such a paradigm to patients with DOC it is necessary to know if the distinction between P3a and P3b is consistent among healthy subjects on a single subject level. We want to examine if the distinction between P3a and P3b is a robust effect, i.e. if it can be found in all healthy subjects of our sample. If the effect is not robust, the procedure cannot be applied to detect a possible response in patients with DOC.

In addition, we applied time-frequency analyses to examine whether or not the frequency dimension adds further information to the detection of voluntary brain activation. We expect delta, theta, and alpha alternations to be related to target-distractor differences [19].

## Results

### ERPs

**Figure 1:**
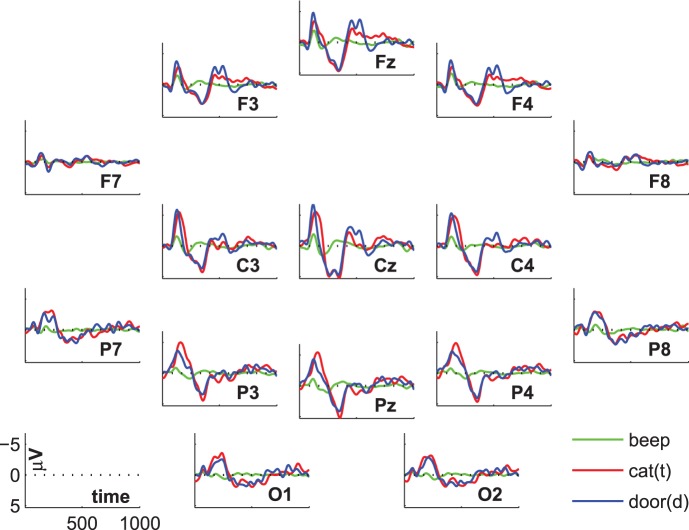
Grand-average ERP.

Figure 1 shows the group-average ERP. We see the oddball-typical deviations between standard and target as well as between standard and deviant tones in the N1, P300, and in a late negative component at 500–600 ms. However, we are specifically interested in the difference between targets and distractors. At parietal electrode positions there is a higher amplitude (i.e. more negative values) for targets compared to distractors at the N1. At central positions, this difference begins after the N1 and becomes visible in the transition to the P300. At frontal electrode positions, the N1 for distractors is higher than for targets. Moreover, there is a larger late negative component (500–600 ms) for distractors than for targets at frontal, central, and parietal electrodes. After the late negative component we can see a positive deviation of the distractors at frontal and central sites, while the targets remain at a more negative level.

**Figure 2:**
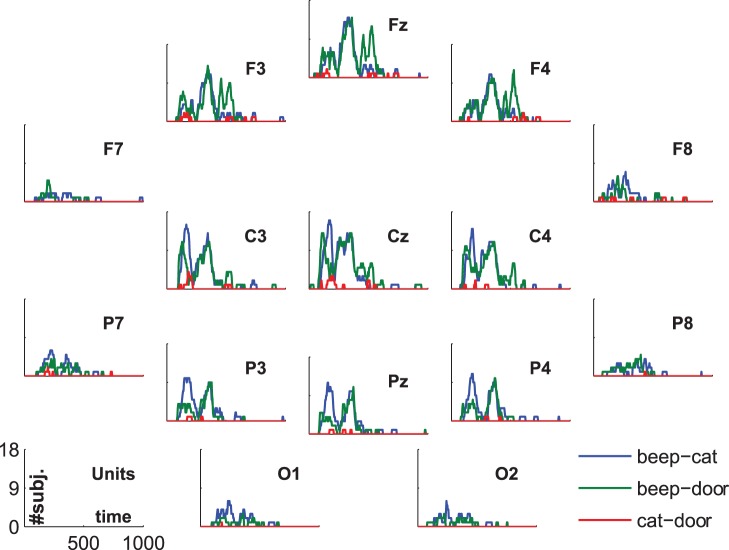
ERP-statistics. Lines indicate the number of subjects showing significant differences between conditions.

The statistical evaluation of the group-average ERPs is shown in Figure 2. The lines represent the numbers of subjects showing significant differences between conditions. In the comparisons between standard and deviant and standard and target we see a high number of participants showing significant differences at the N1, the P300, and the late negative component. The comparison target vs. distractor is of special interest. There are two small increases in number of subjects showing a difference. The first small peak can be found around 250 ms at central positions. The later peak at about 600 ms shows up frontally.

Note that the number of subjects refers to the subjects showing a significant difference at the same instance of time. That is, there can be more subjects showing differences in the time-range of the P300, but these differences do not overlap in time. This may happen if the P300 occurs earlier in one subject than in another subject; e.g. if one subject shows the difference between targets and distractors at the peak of the P300 while the other subject shows the difference immediately after the peak.

In fact, all subjects show a significant difference between targets and standards, as well as between distractors and standards in some subsegment of the time-span between 300 and 400 ms. This effect was most extended in time over subjects on electrode Fz (with similar high values at electrode Cz), for the comparison targets vs. standards with one subject showing no significant difference at this electrode (but rather on electrode C3) so that the binomial significance for electrode Fz was 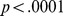. The comparison of distractor vs. standard yielded similar results. The longest effect was found on electrode Fz, which yielded almost the same value as electrode Cz; all subjects showed significant differences in this time-range and at this location (binomial significance 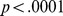). In sum, all subjects showed a P300 effect.

In the group-average ERP, while we see a prominent P300, we see no classical distinction between P3a and P3b. We assessed the significant difference between 200 and 400 ms for the comparison cat(t) vs. door(d) and found that 10 subjects remained without a significant difference. This result indicated a significance on the group level (binomial significance 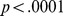). The number of subjects without a significant difference dropped to 6 when we included also the time-frame of the N1, i.e. applied a more rigorous time-range from 150 to 450 ms. The location of the difference varied across subjects, but was pronounced at electrodes Cz, C3, C4, and Pz.

The difference between targets and distractors in the late negative component between 450 and 750 ms was significant in 9 subjects (binomial significance 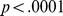), and was most extended in time over central positions (Cz, C3 and C4). Summing up the statistical significance in the time-range of the N1, the P3a/b and the late negative component, 2 subjects remained without a significant difference between targets and distractors.

**Figure 3:**
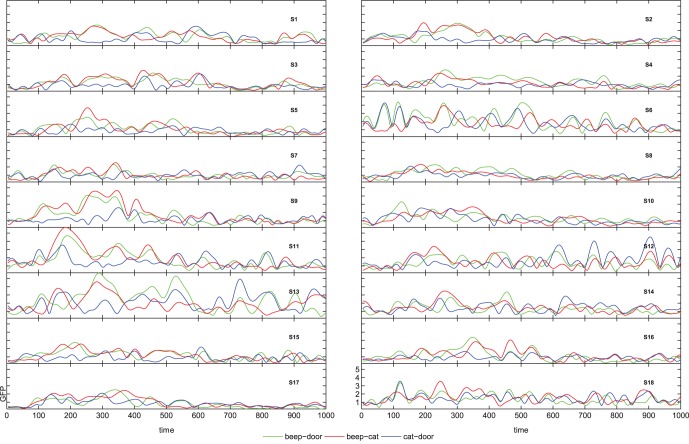
Global field potentials for the difference-waves for all three comparisons.

**Figure 4:**
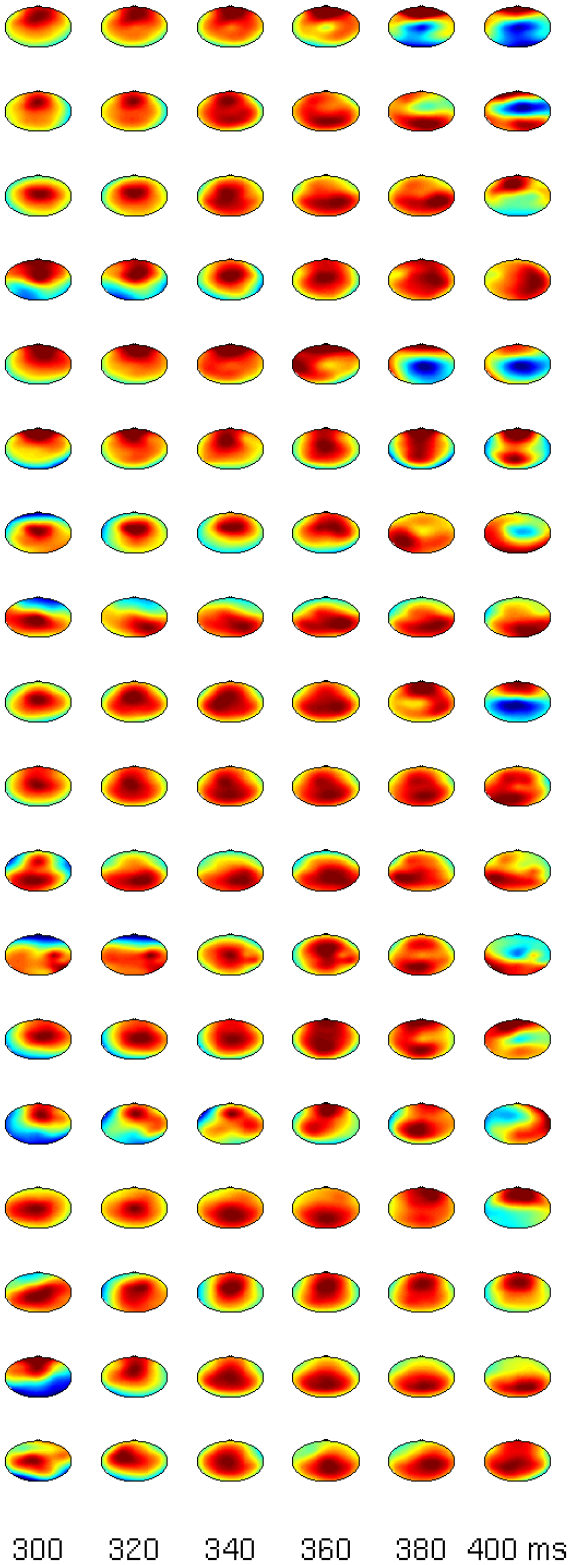
Scalp maps for all subjects (each row represents one subject) between 300 and 400 ms for the comparison target minus standard.

**Figure 5:**
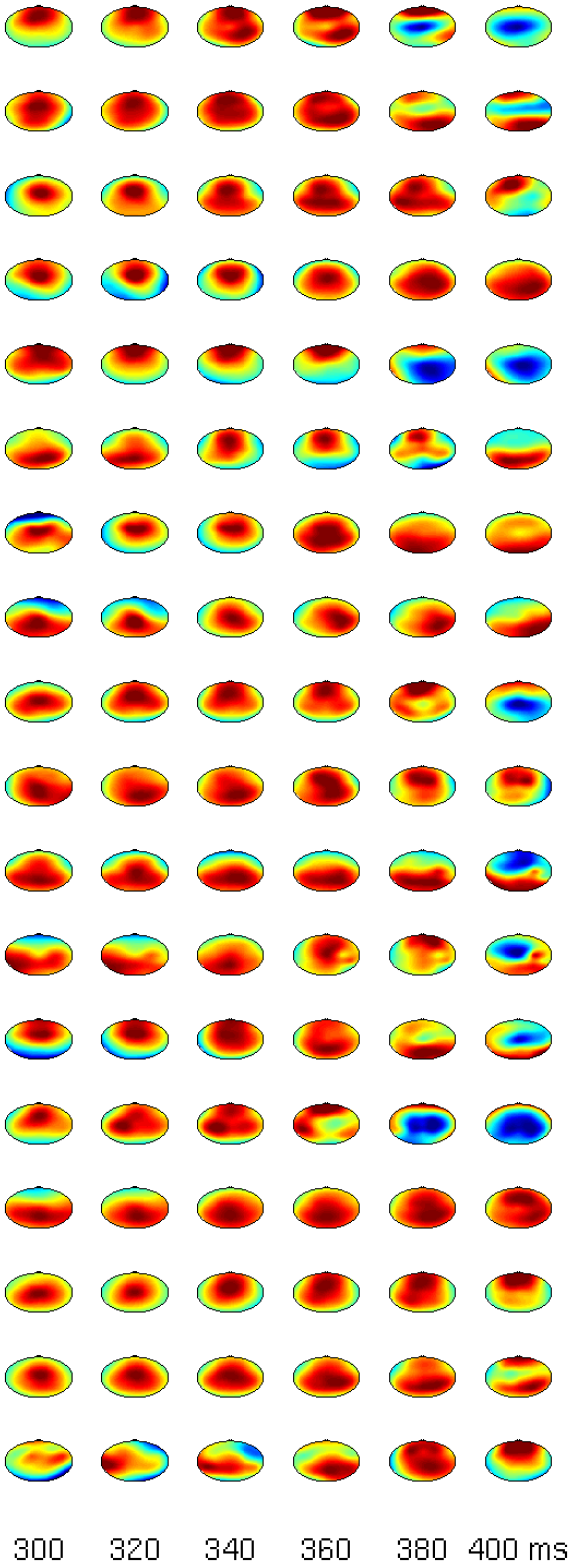
Scalp maps for all subjects (each row represents one subject) between 300 and 400 ms for the comparison distractor minus standard.

**Figure 6:**
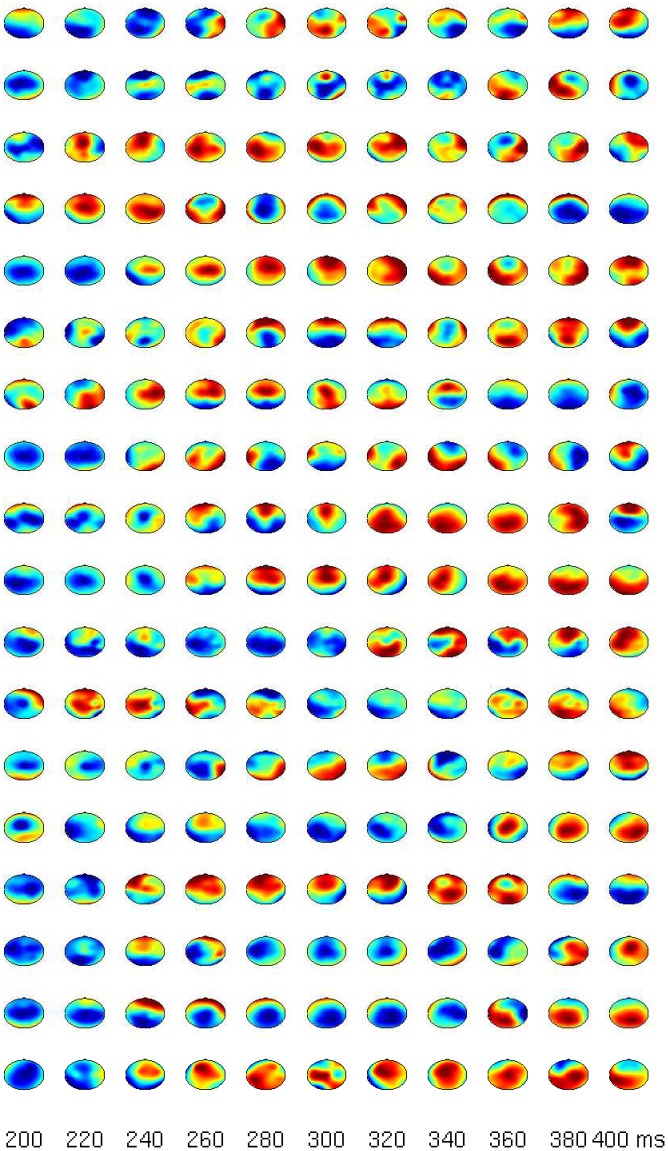
Scalp maps for all subjects (each row represents one subject) between 200 and 400 ms for the comparison cat minus door.

The statistical evaluation of the comparison target vs. distractor suggests that there is a distinction between P3a and P3b in some subjects. The inter-individual variance becomes evident in global-field-potentials (GFPs) in Figure 3. Even tough the effects in the N1 and the P300 for targets/distractors vs. standards are rather consistent across subjects, not all participants show marked increases in the comparison of targets vs. distractors. Most importantly, the blue line (comparison targets vs. distractors) is relatively flat in certain subjects (e.g. S4). To show that the P300 is also spatially consistent across subjects we refer to Figures 4 and 5, which show subsequent scalp maps with a prominent P300 (i.e., a red spot) for all subjects. In contrast, Figure 6 shows a heterogeneous picture for the comparison target-distractor.

For an overview of which subjects showed significant results in most of the mentioned effects and those who showed very little see Table 1.

**Table 1:** Summary of effects for individual subjects.

	subjects																	
effect	1	2	3	4	5	6	7	8	9	10	11	12	13	14	15	16	17	18
**ERP-results**																		
t-s Fz 0.3–0.4 s	×	×	×	×	×	×	×		×	×	×	×	×	×	×	×	×	×
d-s Fz 0.3–0.4 s	×	×	×	×	×	×	×	×	×	×	×	×	×	×	×	×	×	×
t-d all 0.15–0.45 s	×		×		×	×		×	×	×	×	×	×	×			×	
t-d all 0.2–0.4 s	×				×	×		×	×	×				×			×	
t-d all 0.45–0.75 s	×	×			×		×			×		×	×		×	×		
**Time-frequency-results**																		
t-s fpc 0.2–0.6 s 1–5 Hz	×	×	×			×	×	×	×	×	×	×	×		×	×	×	
d-s fpc 0.2–0.6 s 1–5 Hz	×	×	×	×	×	×		×	×	×	×	×	×		×	×	×	
t-s po 0.5–1 s 7–9 Hz	×	×	×				×					×	×		×			×
d-s po 0.5–1 s 7–9 Hz			×												×			
t-s all 0.7 s 1–2 Hz											×				×			
t-s all 0.9 s 7–13 Hz												×						×
d-s all 0.9 s 7–13 Hz															×			
t-d all 0–1 s allHz			×							×								

Significant effects in each subject are indicated with an x; effects are named with the following abbreviations: *t-s* = target vs. standard; *d-s* = distractor vs. standard; *t-d* = target vs. distractor; in ERPs, electrode Fz or all electrodes (*all*) where evaluated; in Time-Frequency results, location was *fpc* = frontal, parietal, and central; *po* = parietal and occipital; and *all* = all locations.

### Time-frequency Data

**Figure 7:**
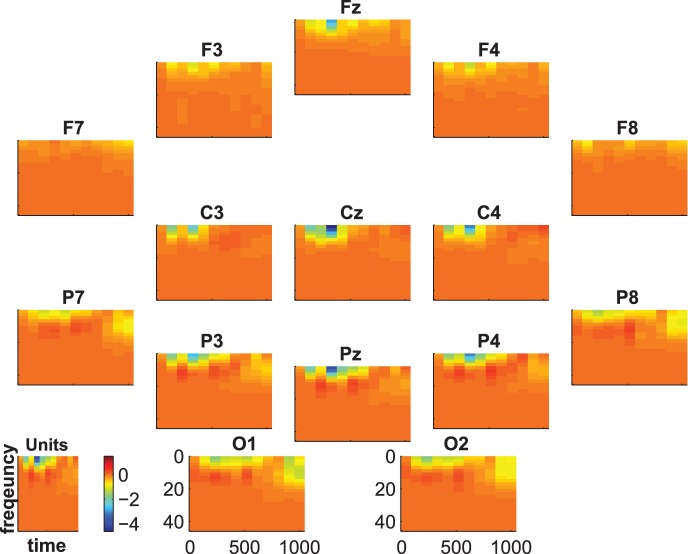
Difference spectrogram standard-target averaged across subjects.

**Figure 8:**
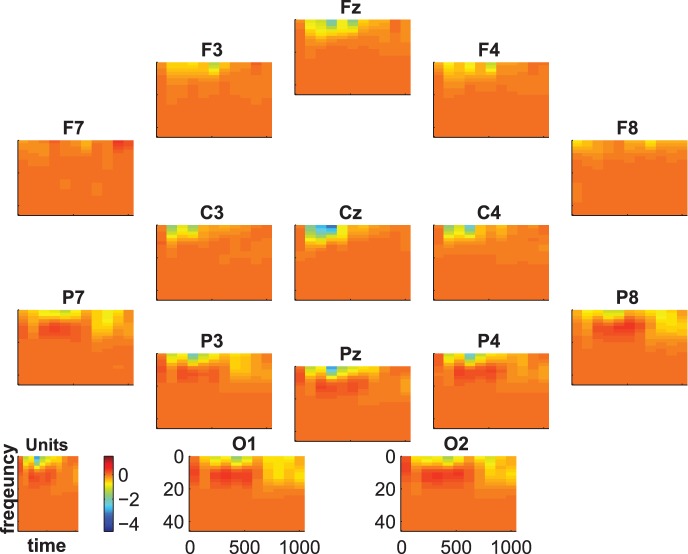
Difference spectrogram standard-distractor averaged across subjects.

**Figure 9:**
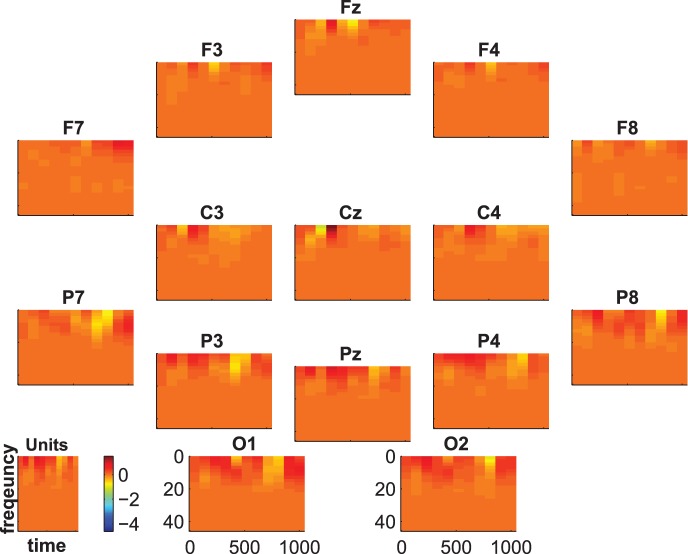
Difference spectrogram target-distractor averaged across subjects.

Figures 7, 8 and 9 show the group-average time-frequency plots for the comparisons beep-cat, beep-door, and cat-door.

The difference spectra standard vs. target/distractor in Figures 7 and 8 show a lower delta-theta activity for standards compared to targets and distractors at about 200–600 ms on frontal, central, and parietal electrodes. In addition, we see a higher alpha-beta activity at the parietoocciptial region for standards compared to targets and distractors at about 300–700 ms.

The difference spectrogram target-distractor is shown in Figure 9. One can see a lower activity spreading from lower frequencies to the beta range at parietal electrodes between 500 and 800 ms for target compared to distractor. Additionally, there is a higher centroparietal delta-theta activity in an early time frame (about 300–500 ms) for targets than for distractors. At C3 and Cz this alternation is preceded by a lower delta-theta activity for targets than for distractors. At F3, Fz, and F4 this alternation is followed by a lower delta-theta activity for targets than for distractors.

**Figure 10:**
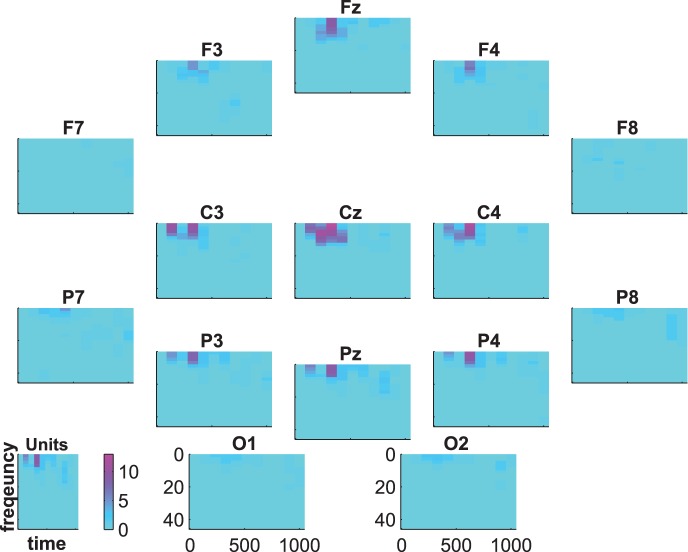
Time-frequency statistics. Coloring indicates the number of subjects showing significant differences between standard vs. target.

**Figure 11:**
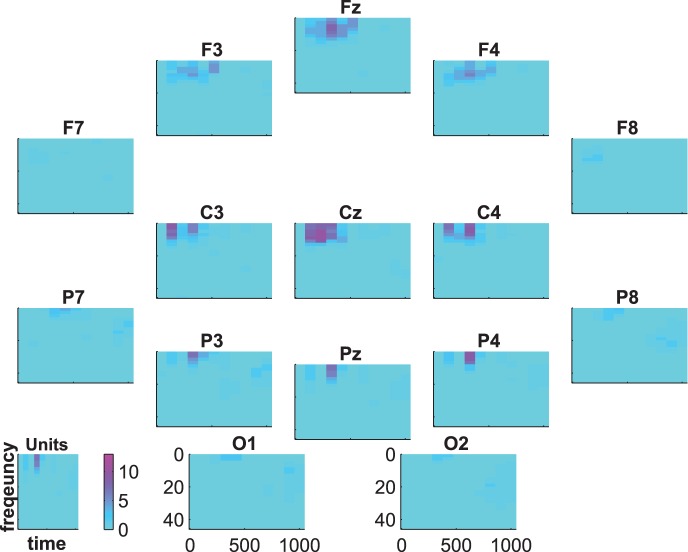
Time-frequency statistics. Coloring indicates the number of subjects showing significant differences between standard vs. distractor.

**Figure 12:**
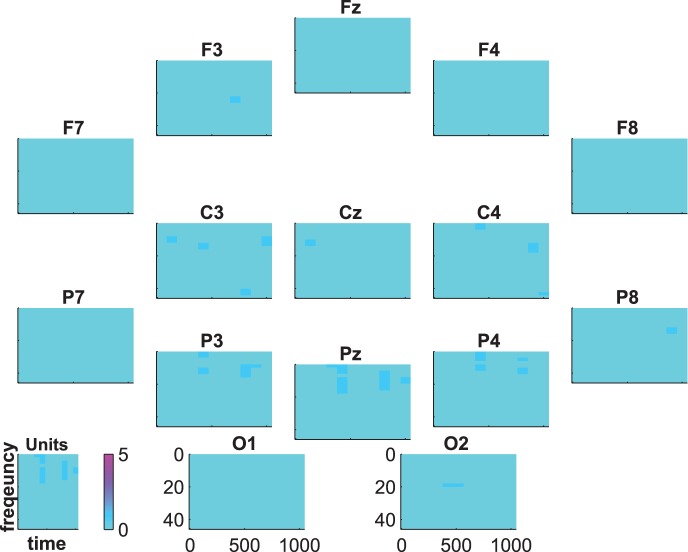
Time-frequency statistics. Coloring indicates the number of subjects showing significant differences between target and distractor.

The statistical evaluation of the group-average time-frequency data is shown in Figures 10, 11, and 12. The coloring represents the numbers of subjects showing significant differences between targets and distractors. That is, the blue regions indicate that there are no subjects with significant differences and the intensity of pink indicates an increasing number of subjects showing significant differences.

Figures 10 and 11 show that the effects we have seen in Figures 7 and 8 are significant in a considerable number of subjects. There was a significant difference between standards and targets and between standards and distractors at frontal, central, and parietal electrodes in the delta-alpha range at about 200–600 ms. This difference is significant in 14 subjects (binomial significance 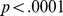) from 1–5 Hz for the comparison standard-target and in 15 subjects (binomial significance 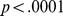) for the comparison standard-distractor. There are fewer subjects showing an alpha effect at about 500–1000 ms at parietal and occipital electrodes. This effect was significant at 7–9 Hz in 6 subjects (binomial significance 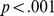) for the comparison standard vs. target and in 2 subjects (no binomial significance 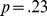) for the comparison standard vs. distractor. At 700 ms there were 2 subjects (no binomial significance 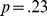) with significant effects in the delta range (1–2 Hz) in the comparison standard-target, and 2 subjects (no binomial significance 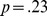) showed a significant effect at about 900 ms in the alpha range in the comparison standard-target; only one subject showed this effect in the comparison standard-distractor (no binomial significance p = .60).

Figure 12 shows the statistical results for the comparison target vs. distractor. At first glance it becomes obvious that the small spots in Figure 9 are significant only in single subjects. This results in no significant group effect (binomial significance 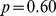). In total, only 2 subjects showed significant differences between target and distractor in time-frequency data. Since these 2 subjects were not the same 2 subjects that showed no significant effect in ERPs, the analysis of time-frequency data did not add further information to the results.

**Figure 13:**
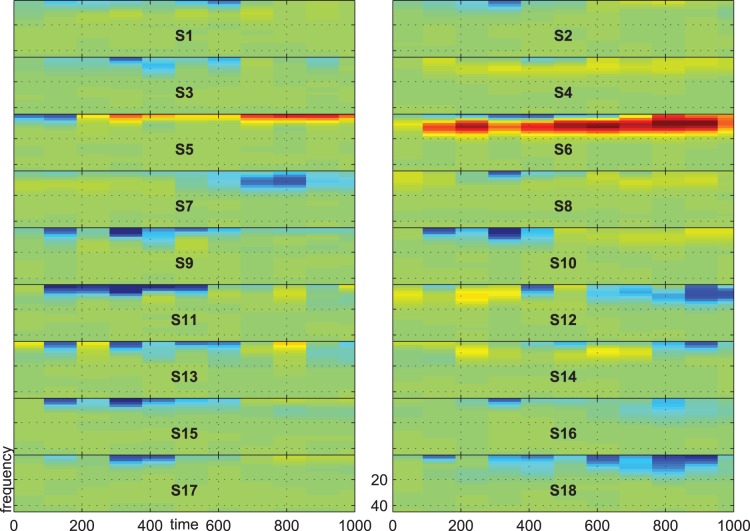
Difference spectrogram for standard minus target on electrode Pz. Scale indicates negative values from −5 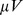 in blue, values near zero in light green and positive values in yellow and red up to 5 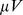.

**Figure 14:**
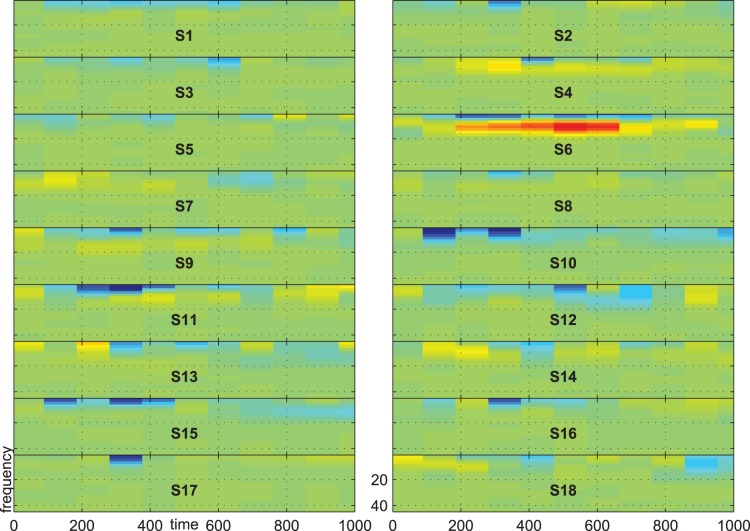
Difference spectrogram for standard minus distractor on electrode Pz. Scale indicates negative values from −5 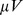 in blue, values near zero in light green and positive values in yellow and red up to 5 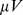.

**Figure 15:**
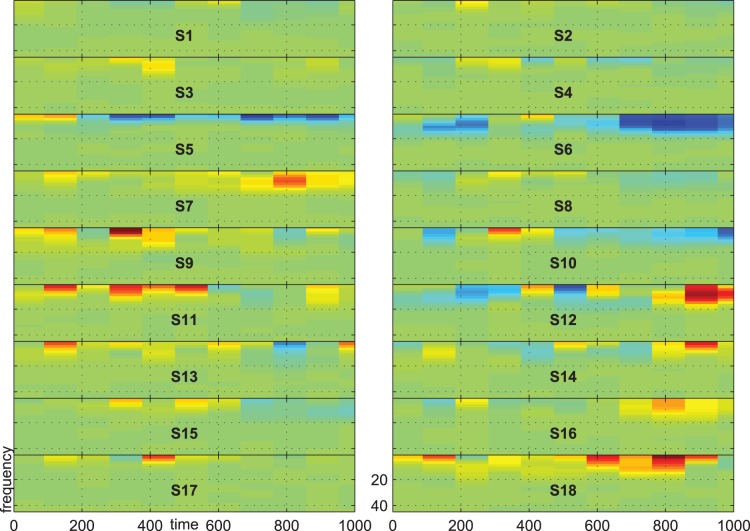
Difference spectrogram for target minus distractor on electrode Pz. Scale indicates negative values from −5 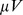 in blue, values near zero in light green and positive values in yellow and red up to 5 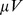.

Figures 13, 14, and 15 show the time-frequency plots of all subjects at electrode Pz. We chose Pz because this location showed a higher difference for the comparison target vs. distractor than other electrodes, e.g. electrode Cz or Fz. The time-frequency plots give evidence for inter-individual difference in response type. For example, subject no. 6 shows higher activity for standard vs. distractor/target in the alpha range, whereas most other subjects show lower activity in these respects.

See Table 1 for a summary of all effects in each subject.

## Discussion

In this work we examined the consistency of effects of voluntary brain activation in an active oddball task. Even though the number of subjects showing a distinction between targets and distractors in the range of P3a/b was significant, it was not robust at all. An effect was found in 8 out of 18 subjects. When taking into account significant differences in the time-range of the N1 and at a late negative component, 2 subjects remained without significant distinction between targets and distractors. The time-frequency results were not useful in distinguishing targets from distractors. Most importantly, analysis of time-frequency data added no information to the detection of voluntary brain activation in this paradigm. Based on these results, we doubt the usefulness of time-frequency- and ERP-analysis of the active oddball paradigm to detect voluntary brain activation in patients with DOC.

The P3a is known to reflect novelty while the P3b reflects target detection [18]. The group-average ERP shows a distinction between targets and distractors in the N1 but no clear distinction between P3a and P3b. Eventually, there is a tendency towards a P3b at parietal electrodes, where the waveform of targets exceeds those of the distractors. Although we see no clear distinction between P3a and P3b in the group average, this does not mean that there is no P3a–P3b complex in any subject. Instead, when analyzing the effect on a single subject level we took into account the inter-individual variance in time and found a significant distinction between targets and distractors in 8 subjects, which were most pronounced over central positions, which is the location where one would expect the P3a to occur [20].

The distinction between P3a and P3b is a relative robust effect. However, the P3b is usually found in experiments when subjects are asked to react to a target by pressing a button. In contrast, our subjects were asked to count the targets silently. It was reported recently that intra-subject variability of reaction times is relatively stable within subjects across trials and influences the amplitude of the P3b [21], [22]. We cannot measure the reaction time of silent counting, but it is very likely that the intra-subject variability in these respects is higher in subjects who show no clear distinction between targets and distractors. A demanding counting task was found to attenuate the amplitude of the P3 [23]. Thus, we could speculate that the intra-subject variability is generally higher in silent counting than when subjects are required to press a button, making the detection of P3b in such experiments difficult.

Lack of control if the subjects were actually performing the task may be considered as another confounding factor. It is possible that some of the subjects that did not show a significant difference in the P3a/P3b period did not perform the task, or did not pay attention to the targets, despite being instructed to do so. In overt-counting tasks, e.g. by asking subjects to press a button whenever they detect a target, the performance of the subject can be correlated with the ERP-components or frequency characteristics. However, this is not an option in unconscious patients, so that it is important to establish reliable task-related brain responses in healthy participants which depend purely on voluntary brain activation.

In addition, it is possible that the semantic nature of our paradigm confounds the distinction of P3a/b in some subjects. We applied cat, door and beep sounds instead of three similar beep sounds with different pitch. The amplitude difference in the range of 300 ms between standard and distractor was found to depend on the nature of the difference [24]. In an experiment with three tasks, Halder et al. [24] found that if target and distractor differ in pitch or frequency, the P300 is generally larger than with amplitude modulation of the stimuli. Most subjects showed best discrimination between targets and distractors when the stimuli differed by pitch. In this study, automated classification was used to distinguish targets from distractors. Most interestingly, while 5 out of 20 subjects showed best classification accuracies among all three tasks above 90%, a further 5 out of 20 subjects showed best classification accuracies below 70%. This proportion of subjects possibly matches the proportion of subjects without a significant difference in our sample, and does support our suggestion that the target-distractor discrimination is not reproducible in all healthy subjects.

The stimuli that were to be counted or ignored in our experiment have different emotional valences (cat vs. door), and the emotional valence may vary between subjects. The emotional valence of the subject’s own name was presented by Schnakers et al. [13]. In their experiment, the subject’s own name and 7 other unfamiliar names were presented in a randomized order. The P300 to subject’s own name was strongly pronounced when the participants were instructed to count the instances of their own name. Subject’s own name also evoked a prominent response when subjects were instructed to passively listen to the stimuli. In contrast, unfamiliar names evoked a P300 when subjects should count these names, but not in the passive condition. It is possible that the emotional valence of the sound of a cat differs between subjects and, therefore, the cat-target stimulus evokes different brain responses compared to the door-distractor stimulus, independent of the instruction to pay attention or to ignore the stimuli. According to Ferrari et al. [25], the amplitude of the P3 reflects stimulus meaning, significance, and novelty. Therefore, a stimulus that may have specific meaning to a subject (sound of a cat or subject’s own name) may result in a higher P3.

By including also the N1 there remained 6 subjects and by including also the late negative component there remained only 2 out of 18 subjects without significant target-distractor distinction. In a visual oddball task which required silent counting of targets [26], 9 out of 14 subjects showed divergence between targets and standards in early components, with a mean latency which corresponded with the P300. In this study, 6 subjects showed a late divergence, with latency similar to our late negative component. There were 3 subjects without significant distinction between targets and standards. Please note that the task of this study did not include a distractor category, and therefore comparisons are limited. However, in both task designs we cannot rule out the possibility that the late negative component may be related to automated processing of semantic content. Thus, we cannot be sure if the significant difference is caused by the brain activation related to counting or to the different processing of stimuli. This distinction between automated processing and voluntary counting is crucial for defining conscious behavior. Thus, this answer has to be answered before applying the task to patients with disorders of consciousness. However, as reported in the study involving a visual oddball task, [26], not necessarily all subjects show significant distinction between stimulus categories. For supporting DOC diagnoses, we believe that it is necessary to choose a task which reliably provokes a voluntary brain response in healthy subjects.

Our study is not the first which documented missing P3a/b effects in single subjects. We found no distinction on group level, but a significant distinction in single subjects. Kotchoubey et al. [27] found the opposite pattern of inconsistency between single-subject data and group-average ERPs. The authors found increased amplitude of the N1 and P3 waves in response to a person’s own name in the group ERPs, but no such response in the individual waveforms of 6 out of 14 healthy subjects, and in 3 out of 5 patients. There are known factors moderating ERP components such as the P300. Schiff et al. [28] reported increasing P300 latencies and decreasing amplitudes with age. In addition, the authors found higher P300 amplitudes in women than men. In addition, the repetition of an experiment leads to longer P300 latencies in the elderly. Therefore, the properties of the examined sample of participants may determine if there is a group-effect or not.

Despite questioning the use of ERP- and time-frequency analysis in an active oddball paradigm to detect consciousness in patients of DOC, our results support the claim for single-subject analysis in EEG. Sophisticated algorithms and elaborated tools pave the way for single-subject statistics as a basic requirement in EEG-research. In the following, we want to describe how our results give evidence for the need of single-subject analysis.

First, a significant effect may be consistent across some subjects with respect to time, frequency and electrode position, but it may differ with respect to direction. That is, subject A may show higher alpha activity for targets compared to distractors, while subject B could show higher alpha activity for distractors compared to targets. If both effects were of similar strength the effect would not be visible in the group-average time-frequency plot. We illustrated this situation in Figure 13, and 14, where one subject shows a higher activity in the alpha range for standards compared to distractors and targets, while the other subjects show an alpha decrease. In our earlier research we reported inter-individual differences in the kind of alpha-response (increase/decrease) during motor-imagery [15], to self-selected music [29], in the classic oddball-task [30], and when listening to subject’s own name [31]. The present results provide further evidence for individual alpha-response types. However, it is important to note that the kind of alpha-response might not be a stable trait, but could rather reflect the state [32] or the developmental stage of a subject. Alpha power can be altered by many variables, e.g. with age [33], and is moderated by a number of pathologies [34].

Second, the strength of effects may vary largely between subjects. There may be 2 subjects in a sample with a large alpha desynchronization at a certain instance of time. This desynchronization could be so strong that it becomes visible in the group-average time-frequency plot. However, 2 subjects with significant effects would only show up slightly on a statistical plot as used in this study. In contrast, there could be 5 subjects with a very small alpha synchronization which was highly reliable across trials, and which would therefore result in a statistically significant effect. Such an effect would not show up in the group-average time-frequency plot but would be visible in the statistical plot. The individual strength of power-changes is only rarely reported. Pfurtscheller et al. [35] reported variable power changes in a motor-imagery task; e.g., one subject showed a band power change of 245%, another subject showed only 21%. To solve this problem one can normalize the data or calculate single-subject statistics. A statistical method like the single threshold permutation test which treats the frequency bands individually has the additional advantage that no normalization of frequency bands is needed. Thus, the problem that alpha oscillations show up with higher amplitudes than gamma-oscillations is handled by the statistical procedure.

Interindividual variance is a problem for both ERPs and time-frequency data, if analyzed on group level. On the background of documented variance, group-level effects cannot be generalized to clinical populations without testing the consistency of the effect in single subjects. The possible value of single trial and single-subject analysis was recognized at least 25 years ago [36]. Recently developed algorithms and freely available software boost the use of single-subject analysis in EEG [37]–[40].

## Conclusions

Before the active oddball paradigm can be applied clinically for detecting voluntary brain activation in patients with DOC a reliable analysis of the response must be established in healthy subjects. We found that the distinction between target and distractor was highly inconsistent between subjects in the ERPs as well as in time-frequency data. Given the results of the present study, we would not recommend analyzing an active oddball paradigm with ERPs or time-frequency representations to assess voluntary brain activation (i.e., active counting of the target) in unresponsive patients. However, a limitation of the study is that we used a rather specific semantic oddball, which may have confounded the results. Future experiments should clarify whether the distinction between P3a and P3b in the ERP shows less inter-individual variability with other stimulus qualities and whether the late negative component can distinguish conscious from unconscious target processing. In addition, examination of other analysis techniques could be promising.

However, our results give further evidence that single-subject statistics are necessary in EEG research. Plotting the ERP and analyzing group effects may be misleading if the consistency across subjects is poor. In functional magnetic resonance imaging it is standard to apply analysis to single subjects as well as on group level. Fast algorithms and freely available tools make single-subject analysis of EEG-data feasible. We believe that future research will rely stronger on single-subject analyses of EEG-data because results obtained on a group level often reflect effects which are only present in a specific subgroup of the tested participants, making general inference to population unwarranted.

## Materials and Methods

### Ethics

Written informed consent was obtained from each subject according to the ethical guidelines of the Declaration of Helsinki. The experiment was part of a larger project which has been approved by the ethics committee of Salzburg.

### Subjects

We recruited 29 healthy subjects, of whom 23 participated in this experiment, 6 participated in a variant of the experiment. Because of technical reasons, 5 subjects were excluded from data analysis. Data collected from 18 high school graduated subjects (age: 20–26 years; mean = 23 years; SD = 1.95; 6 male) was analyzed. None of the participants reported any history of neurological or psychiatric diseases, and they were not receiving any psychoactive medication.

### Experiment

The experiment was split in two parts, each preceded by an instruction (duration = 18 sec) which was presented via headphones. In both parts an equal number of stimuli were presented. A frequently presented (total: 300 trials) beep-tone served as standard. Additionally, the rarely presented (total: 50 trials each) sound of a cat and the sound of a doorbell served as deviants. Each deviant was preceded by 2–7 standards. The instruction asked the subjects to count the sound of the cat and to ignore all other stimuli. Each trial lasted 1500 ms and a variable timespan of 34–480 ms to avoid expectancy effects.

### Data Registration

EEG-Data was recorded using a BrainCap with a 10–20 system and a BrainAmp (Brain Products GmbH, Germany) 16-bit ADC amplifier. The sampling rate was 250 Hz. Of the 32 recorded channels, 2 were used to monitor the left and right horizontal electrooculogram. One was used to monitor lower-site vertical electrooculogram. Two were positioned at the mastoids for re-referencing purposes to remove the bias of the original reference, which was placed at Fcz. The other electrodes were Fp1, Fp2, F3, F4, C3, C4, P3, P4, O1, O2, F7, F8, T7, T8, P7, P8, Fz, Cz, Pz, FC1, FC2, CP1, CP2, FC5, FC6, CP5, and CP6. Data analysis was conducted for data collected from the electrodes F3, F4, C3, C4, P3, P4, O1, O2, F7, F8, P7, P8, Fz, Cz, and Pz. Impedances were kept below 10 k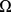.

### Data Preparation

Data pre-processing was done with Brain Vision Analyzer (Version 1.05.0005, Brain Products GmbH). First, mastoid electrodes were used to build a new averaged reference for all other channels. The average of Fp1 and Fp2 was used as a reference for the lower-site vertical electrooculogram to obtain a bipolar vertical signal. To reduce noise, Butterworth Zero Phase Filters from 1 to 48 Hz (time constant 0.1592 s, 48 dB/oct) were applied.

Independent component analysis (ICA) was applied, since this procedure has been shown to effectively detect, separate, and remove ocular, muscular, and cardiac artifactual sources in EEG data [41]–[43]. The ICA was calculated on all channels, including the prepared electrooculographic channels. After visual inspection of the ICA components, those components containing ocular or muscle artefacts were determined and removed by performing the corresponding ICA back-transformation.

An automatic data inspection was carried out in order to exclude remaining artefacts. Maximal allowed voltage step per sampling point was 50 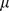
V (exceeding values were excluded with a surrounding of ±100 ms); maximal allowed absolute difference on an interval of 200 ms was 200 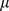
V and lowest allowed absolute difference on an interval of 100 ms was 0.5 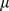
V (exceeding values were excluded with a surrounding of ±500 ms).

To perform frequency analysis on individual trials, data was segmented into 2.2 sec epochs for each trial. These segments started −600 ms before begin of the stimulus and ended 1600 ms after begin of the stimulus. The preprocessed segments were exported into a generic data format and imported to MATLAB® (The Mathworks). All of the following steps (analysis of ERPs and time-frequency data, and the respective statistics) were carried out in MATLAB.

### Event Related Potentials (ERP)

All segments were band-pass filtered from 1–20 Hz and then truncated from 0 to 1000 ms. These segments were then analyzed using the statistical methods described in the Statistics section.

For the purpose of plotting graphs, the means of all trial-types were built. The ERPs with three lines for the three trial types (target, distractor, and standard) were drawn. To illustrate inter-individual variance, global field potentials (GFPs) were computed and drawn for each subject individually. In addition, difference scalp maps were plotted for each 20 ms step between 300 and 400 ms for the comparison between standards and distractors or targets, and between 200 and 400 ms between targets and distractors. The different time-ranges were chosen to catch the P300 between 300 and 400 ms and the earlier occurring P3a together with the P3b between 200 and 400 ms.

### Frequency Analysis

The 2.2 sec segments were transformed into time-frequency-domain by applying a short time Fourier transform on 128 ms subdivisions of the segments using a Hamming window. The segments overlapped for 64 ms. The frequency resolution was chosen to be 2 Hz, beginning from 1 Hz up to 45 Hz. For each trial the baseline was estimated by calculating the mean of the estimate of the short-term, time-localized frequency content at the subdivisions from −200 ms to 0 ms. This baseline was subtracted from the values from time 0 ms to 1000 ms. The segments were then truncated from 0 ms to 1000 ms. These time-frequency representations for all trials were then analyzed with the statistics described in the Statistics section.

For graphical purposes, the means of all target-, distractor-, and standard-trials were built. Then, the mean of targets was subtracted from the mean of standards, the mean of distractors was subtracted from the mean of targets, and the mean of distractors was subtracted from the mean of targets. The values of the 3 difference-matrices were normalized to a range from the minimum to the maximum of all 3 difference-matrices and displayed as images. To illustrate inter-individual variability these difference-plots were given for a single electrode position, individually for each subject.

### Statistics

Single threshold permutation tests as described by Nichols and Holmes [44] for functional neuroimaging were implemented in MATLAB® and performed on a single subject level. Similar approaches for the EEG are described by Maris and Oostenveld [39] and Koenig et al. [38]. The single threshold permutation test works as follows:

1. The trials of two conditions are collected in a single set.
2. This set is shuffled and partitioned into two sets which have the same size as the original trial sets of the two conditions. However, these random partitions now include trials from both conditions.
3. A test statistic, in our case a t-test, is calculated for each data point (i.e., each time-electrode or each time-electrode-frequency combination) on this random partition. The most negative and the most positive of the resulting t-values are recorded.
4. Steps 2 and 3 are repeated for 1000 times and a histogram is constructed of the 1000 recorded t-values separately for the most negative and the most positive t-values.
5. A t-test is calculated on the original trial sets (that is, each trial set contains only trials from one condition). Based on the histograms calculated in step 4, the proportion of random partitions that resulted in a more extreme (i.e. more negative or more positive) t-value than the observed one is calculated. This proportion is the p-value of the permutation-t-test.
6. If the p-value is smaller than the critical alpha-level we conclude that the test result is significant.

This test was applied to all data points, i.e. to each time-point at each electrode of the ERPs, and to each time-frequency point at each electrode in time-frequency maps. Note that this usually yields the multiple-comparisons problem. In fact, the described permutation t-test is designed to deal with this problem by recording all t-values of all t-tests (i.e. all t-tests for each time-(frequency)-electrode-combination) and calculating the p-value based on the distribution of these t-values. Thus, the critical alpha level directly indicates the probability that the difference happened by chance, since the calculated p-value indicates the probability of more extreme results under random conditions.

An additional advantage of this test is that no further normalization is needed. By applying t-tests individually to each time-frequency point, the frequencies are all treated individually. The differences between two conditions may be larger for the alpha range than the gamma range, for example, but are normalized by calculating the t-values of these differences.

Such a single threshold permutation t-test was applied to all possible pairs of conditions; that is, beep vs. cat(t), beep vs. door(d), and cat(t) vs. door(d). Because we carried out three permutation t-tests, a corrected critical alpha level of 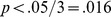 was applied. The corresponding t-values for this critical alpha level according to the above described histograms are determined individually for each subject and were on average across subjects 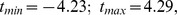 for beep vs. cat(t), 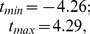 for beep vs. door(d), and 
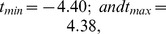
 for cat(t) vs. door(d) for ERPs and 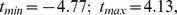 for beep vs. cat(t), 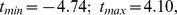 for beep vs. door(d), and 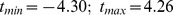 for cat(t) vs. door(d) for time-frequency data.

The spectrogram-statistics were finalized by counting the number of subjects showing significant t-values for each time-frequency step and each electrode position. Statistics for ERPs were done analogously in the dimensions of time and electrode position. The resulting numbers were plotted to give an estimate of the consistency of the effect across participants.

However, this illustration represents only the numbers of participants showing simultaneously significant effects. We counted the numbers of significant time-bins in certain time-ranges to see if an effect occurred in each of the participants, e.g. between 300 and 400 ms for the comparisons with the standards (beep, i.e. to catch the P3 effect) and between 200 and 400 ms for the comparison cat(t) vs. door(d) (i.e. to catch the earlier P3a together with the P3b) in the ERP for each participant, for each electrode and each comparison individually. Then we evaluated if there were subjects without significant time-bins. We examined which electrode showed the highest sum across subjects in each comparison and determined the range of significant time-bins across participants at this position.

In order to evaluate the number of subjects showing significant differences on group level we applied binomial tests (e.g. as used in [37]) by use of the free Matlab function myBinomTest with n = 18 (number of subjects), p = 0.05 and s expressing the number of subjects with a significant p-value as determined with the individual permutation tests. We chose to apply one-sided tests because we wanted to test if the observed number was greater than the expected number of successes.
